# Differential effects of ongoing EEG beta and theta power on memory formation

**DOI:** 10.1371/journal.pone.0171913

**Published:** 2017-02-13

**Authors:** Sebastian Scholz, Signe Luisa Schneider, Michael Rose

**Affiliations:** 1 Department of Systems Neuroscience, University Medical Center Hamburg Eppendorf, Hamburg, Germany; 2 Department of Psychology, Carl von Ossietzky University of Oldenburg, Oldenburg, Germany; Universitair Medisch Centrum Groningen, NETHERLANDS

## Abstract

Recently, elevated ongoing pre-stimulus beta power (13–17 Hz) at encoding has been associated with subsequent memory formation for visual stimulus material. It is unclear whether this activity is merely specific to visual processing or whether it reflects a state facilitating general memory formation, independent of stimulus modality. To answer that question, the present study investigated the relationship between neural pre-stimulus oscillations and verbal memory formation in different sensory modalities. For that purpose, a within-subject design was employed to explore differences between successful and failed memory formation in the visual and auditory modality. Furthermore, associative memory was addressed by presenting the stimuli in combination with background images. Results revealed that similar EEG activity in the low beta frequency range (13–17 Hz) is associated with subsequent memory success, independent of stimulus modality. Elevated power prior to stimulus onset differentiated successful from failed memory formation. In contrast, differential effects between modalities were found in the theta band (3–7 Hz), with an increased oscillatory activity before the onset of later remembered visually presented words. In addition, pre-stimulus theta power dissociated between successful and failed encoding of associated context, independent of the stimulus modality of the item itself. We therefore suggest that increased ongoing low beta activity reflects a memory promoting state, which is likely to be moderated by modality-independent attentional or inhibitory processes, whereas high ongoing theta power is suggested as an indicator of the enhanced binding of incoming interlinked information.

## Introduction

The ability to encode new information into long-term memory is essential in every human's day-to-day life. Neuronal processes at learning that are associated with subsequent remembering in contrast to subsequent forgetting are commonly summarized in the literature as subsequent memory effects (SMEs) [1]. Various methods have been employed to investigate physiological structures and processes that give rise to these effects: Electroencephalography (EEG; e.g. [1–4]), magnetoencephalography (MEG; (e.g. [1–3, 5]), functional magnetic resonance imaging (fMRI; e.g. [6–8]), and intracranial-electroencephalography (iEEG; e.g. [9–11]).

Early seminal studies investigated SMEs with respect to post-stimulus brain activity (e.g. [1–3]), but since ongoing neural fluctuations shortly before the occurrence of an event were shown to have an impact on various aspects of cognitive functioning (e.g. [12–14]), recent research has also started to address the role of pre-stimulus brain activity in the formation of memory for the upcoming stimulus. Previous studies using event-related potentials (ERPs) revealed that frontal negative activity around 250 ms prior to stimulus presentation predicted successful encoding of visually presented words [15]. Other research investigated SMEs by analyzing time-frequency decomposed neurophysiological data. These studies typically found increased pre-stimulus activity in the theta frequency band to be associated with successful later retrieval of an item [4, 5, 16], with the source of this activity being localized in the medial temporal lobe structures [5, 7]. Other studies also report pre-stimulus SMEs in the alpha (9–12 Hz) [6, 9, 17], low beta (13–17 Hz) [18, 19], and high beta (18–30 Hz) [20] frequency ranges. The majority of investigations announced the to-be-remembered stimulus by a cue, either explicitly by a specific item signaling the occurrence of a stimulus or implicitly by a steady fixation time preceding the stimulus itself. Therefore, in those studies, it has not been possible to disentangle, whether the observed neural activity was related to preparatory processes induced by the cue or to ongoing, fluctuating neural activity. Salari and Rose [18] on the other hand, investigated ongoing neural activity and revealed an increase in ongoing EEG theta and low beta activity to be associated with successful memory encoding. These effects were largest at frontal and temporal electrodes, starting about one second before stimulus onset. In a second experiment, these findings were used to take a deeper look into the functional relevance of pre-stimulus activity for subsequent memory formation by utilizing the detected neural oscillations in a brain computer interface (BCI). The presentation of a stimulus was adaptively timed to points at which the power of the frequency of interest was either high or low [21]. Stimuli presented in a state of increased beta power were more likely to be remembered later than those presented in a state of decreased beta power, suggesting a functional role of activity in the low beta range in memory formation. In contrast, no functional role of theta activity could be established by this approach. Further, Schneider and Rose [19] compared pre-stimulus beta activity during intentional and incidental encoding of information and revealed that the mere intention to encode a stimulus increased the pre-stimulus SME in the lower beta band, suggesting that this effect might reflect encoding-specific processes.

Up to this point, it has not been conclusively clarified whether these pre-stimulus SMEs reflect general memory-promoting states or whether they are specifically linked to cognitive processes that are only related to certain aspects of memory formation. For example, the general level of attention or motivation can modulate consecutive processing and is reflected in pre-stimulus activity. Task independent attention usually is reflected in alpha band activity, but can also affect the low beta band [22]. A frequently discussed function of this activity is inhibitory processing of the task irrelevant brain regions [22–24]. Activity in low beta frequencies has been further associated with predictions of upcoming stimuli and maintaining a defined level of processing [25–27] and also with direct memory related preparation processes [23]. Lower beta activity might prepare the memory system for later remembering by influencing one or more of these memory-related cognitive processes. The direct memory related function of beta- band activity for item memory was also supported by the previous BCI study [19]. In contrast, pre-stimulus theta activity might influence memory performance more directly by enhancing associative memory formation in particular for item-item associations or source memory [4, 28]. A dissociation of the role of different pre-stimulus effects in different frequency bands would help to differentiate unspecific pre-stimulus effects from more directly memory related oscillatory activity.

One interesting and so far rather neglected question is whether such SMEs depend on the modality of the to-be-encoded stimulus. While pre-stimulus SMEs have been reported repeatedly for visually presented verbal [4, 5, 9, 16] and pictorial stimulus material [18, 19, 29], SMEs for aurally presented stimuli have rarely been examined. In previous ERP research, SMEs related to auditory stimuli showed the same frontal negative peak around 250 ms prior to stimulus onset as visual stimuli [30]. However, at this point, it is not clear whether similar ongoing oscillations in the theta and lower beta frequency bands are related to successful encoding in aurally presented stimuli as have been reported for the visual domain. The present study aimed at filling this gap by comparing oscillatory pre-stimulus SMEs for visually and aurally presented words.

Some studies looked more deeply into the influence of pre-stimulus oscillations on the quality of the memory trace which is built up during encoding by dissociating recollection (detailed memory for the item and the situation in which it was encoded) from familiarity (having a rather vague feeling of memory for the item but no additional context memory) and found increased theta power to be predictive of subsequent recollection of items [30]. Further, it has been shown that later recognition memory but not free recall performance could be predicted by pre-stimulus theta power during encoding [4], suggesting that pre-stimulus theta activity might reflect a beneficial state for the encoding of stimulus information by enhancing the ability to form associative memory traces. Interestingly, however, no association between pre-stimulus theta activity and shallow or deep encoding strategies has been found [5, 29], which indicates that the potential function of pre-stimulus theta SMEs to enhance associative memory processes does not depend on conscious strategy usage. The present study aimed to shed further light on the relevance of ongoing oscillatory activity on qualitative aspects of memory by disentangling memory for the item itself from memory for an associated context picture.

### The present research

The present study investigated pre-stimulus SMEs in different sensory modalities by employing a within-subject design with visually and aurally presented stimuli in a memory task. During the incidental encoding phase of the study, written and spoken words were presented in a block design. To investigate qualitative aspects of memory, the stimuli were accompanied by visual context pictures. The background pictures can be used to test for an associative memory aspect of the stimulus with the context in which it was presented (source memory). The retrieval phase consisted of a recognition memory task, in which old and new stimuli were intermixed. While old stimuli were presented in the same modality as during encoding, new stimuli were presented in a random modality. Both memory for the item itself as well as for the accompanying context picture had to be indicated.

With regard to pre-stimulus SMEs, correct recognition in the visual modality was hypothesized to be accompanied by increased pre-stimulus low beta and theta activity in frontal and temporal electrodes -1000 to 0 ms before stimulus onset. This would be a replication of prior results by Salari and Rose (Experiment 1, [18]). Since ERP research revealed similar effects for spoken and written words [30], the same pattern was expected for time-frequency decomposed data in the present investigation. In addition, increased theta power was expected for successful source retrieval, as this frequency has been related to qualitative, associative aspects of memory [4, 31].

## Materials and methods

### Participants

The sample of participants consisted of 19 persons (10 females) within an age range of 19–33 years (M = 26.21, SD = 3.1). All participants were students from one of the universities in Hamburg (Germany), had normal or corrected-to-normal visual and auditory acuity, and were right-handed. Participants were paid in exchange for their participation in the experiment. The study was approved by the ethics committee of the German Psychological Association. Participants provide their written informed consent and the documents were deposited in a locked place (according to the approved procedure).

### Stimuli

The pool of words for the study consisted of 300 German nouns which were 3–8 letters long. The words were taken from the SUBTLEX database that includes the 10,000 most common words from subtitles of movies and TV shows translated from English to German [32]. Out of these 300 words, 200 words were randomly drawn for each participant and presented during the encoding phase of the experiment. Four lists, each containing 50 words, were randomly created from these 200 words. Two lists were presented visually (written in font Helvetica, size 30, color black), the remaining lists aurally, and the order of the lists was randomized. The auditory words were recorded from a male adult speaker in plain German and played via loudspeakers with the same volume for all participants. In the background of the words, pictures were presented, which were high quality photographs of either an office, a kitchen, a forest, or a beach (dimensions 800x600 pixels).

### Design and procedure

Since the aim of the present experiment was to investigate incidental learning mechanisms, participants were not instructed to remember the stimuli at the beginning of the experiment. Instead it was explained that the aim of the study was to investigate the differences between visual and auditory processing. Participants filled out a short demographic questionnaire, were properly seated in an electrically shielded and sound-proofed chamber, with one meter distance from the computer screen, and were connected to the EEG system. The experimental paradigm was presented on a regular computer screen using MATLAB (Version R2013a) with the Psychophysics-Toolbox (Version 3.0.11) (http://psychtoolbox.org/) [33–35].

#### Encoding phase

**Fig 1:**
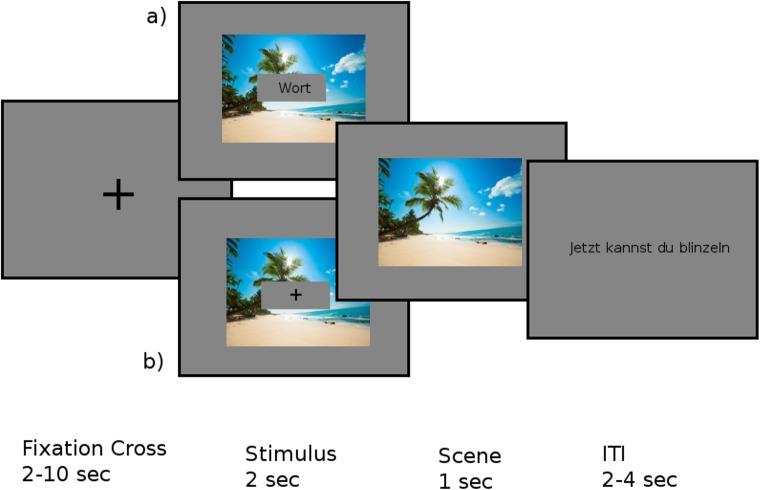
Design of the encoding phase. Dependent of the word list, either a) a visual Item or b) an auditory item were presented in each trial.

In the encoding phase, participants were presented with the four lists of 50 words each, two of which were presented visually, the other two aurally. Participants were tasked with deciding whether each word represented something animate or inanimate. The experiment started with the introductions and one practice trial per modality. At the beginning of each word list, the modality of the following list was indicated. The structure of each trial is depicted in Fig 1. The written words were presented in the center of the screen on a gray rectangle and were shown for two seconds. The average duration of the spoken words was one second. In order to keep presentation durations of the two modalities as similar as possible, the spoken words were accompanied by a fixation cross on top of a gray rectangle presented in the center of the screen for two seconds.

Each to-be-remembered stimulus was preceded by a fixation cross, which varied in duration between two and ten seconds. This was done in order to make the occurrence of a stimulus as unpredictable as possible to be able to study un-cued, ongoing brain activity. In the background of the stimulus, one of the background pictures was displayed. One picture was consistently presented for a complete list of 50 words and therefore served as a context for the later test of the source memory aspect. The pictures were randomly assigned with the lists for each participant. Participants had to make the animacy judgments by pressing buttons on a keyboard with the left and the right index fingers. The key mapping was counterbalanced across participants, and participants were given a maximum of two seconds to respond. After two seconds the word/fixation cross disappeared but the picture remained visible for one additional second. After each trial, there was an inter-trial-interval (ITI) with a variable duration between two and four seconds before the next trial started. Following each word list, a break of one minute was included. On average, the encoding phase lasted 45 minutes.

#### Distraction phase

The distraction phase started with a mental arithmetic task, in which participants had to count backwards from 300 to zero in steps of seven, which was followed by a break of ±5 minutes before the recognition phase began. In total, the delay between encoding and recognition lasted 15 minutes for all participants.

#### Recognition phase

Finally, a surprise recognition task had to be completed in which all 200 items that were previously presented during the encoding phase were once again presented, intermixed with the remaining 100 words from the word pool. The order of the words in the recognition task was random. Modality of old items was kept the same as during encoding phase. Half of the new items were presented visually and the others aurally.

**Fig 2:**
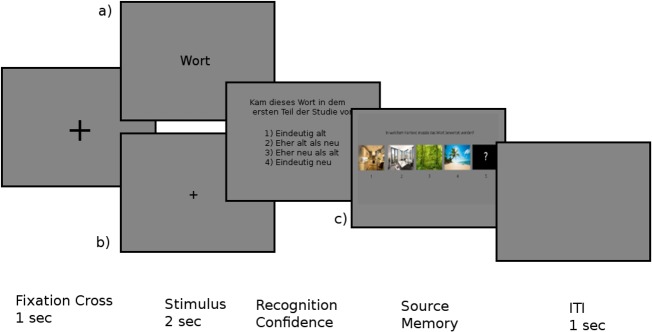
Design of the retrieval phase. In each trial, either a) a visual item or b) an auditory item **was** presented. c) was only presented when recognition confidence was answered with either option 1 (certainly old) or option 2 (presumably old).

The progress of a trial in the recognition phase is depicted in Fig 2. Each trial started with a fixation cross that was shown in the center of the screen for one second. Next, a word was presented that was either old or new and either written or spoken. Written words were once again presented for two seconds in the center of the screen and spoken words were again accompanied by a two-second long fixation cross in the center of the screen. After the presentation of the stimulus, participants had to decide whether the item was old or new. They had to use a 4-point confidence scale, ranging from very certain that an item is old to very certain that an item is new. If participants responded that an item was old, they had to decide which picture was displayed as the background of the item during the encoding phase by a button press, choosing either one of the four pictures or the option that the context is not remembered, reflected by a question mark. Finally, there was a one-second long ITI before the next trial began. Additionally, after every 50 trials, a break of one minute was included. On average, the recognition phase lasted for 35 minutes. Timing of all answers during this part of the study was self-paced, and reaction times were collected.

### EEG data collection

EEG was measured with 60 active electrodes at locations based on the extended 10–20 system (ActiCap, Brain Products, Gilching, Germany). All electrodes were online referenced to FCz. Additionally, four electrodes for vertical and horizontal EOG from above versus below the left eye and from the outer canthi of the eyes were used for detection of eye movements. All impedances were kept below 20 kΩ. The EEG signal was recorded with BrainVision Recorder. During data digitization and amplification, an online filter with a low cut-off frequency of 0.53 Hz was applied. Data were sampled with a sampling frequency of 250 Hz, and all following EEG analyses were performed with MATLAB using Fieldtrip toolbox (http://www.ru.nl/fcdonders/fieldtrip) [36].

### EEG data preprocessing and time-frequency decomposition

EEG data were separated between six conditions in total. Trials involved either aurally or visually presented stimuli, which were either subsequently remembered or forgotten, and remembered trials were split up into those with correct and incorrect source memory. Trials were epoched around the onset of the word with an interval of two seconds pre-stimulus and one second post-stimulus. Next, data were filtered with a bandpass filter using 0.3 Hz and 100 Hz as passband settings, re-referenced to the average of all channels without the EOG electrodes, and baseline-corrected for the complete length of the epoch. Afterwards, the FieldTrip-based automatic artifact detection based on z-scores was applied [36]. In a first step, in order to detect spike artifacts, this procedure removed all trials, in which the cumulative z-scores exceeded the threshold of 20. In a second step, ocular artifacts were addressed by applying the same detection procedure on HEOG and VEOG electrodes with a threshold of a z-score of 4. On average, 74.2% (SEM ± 1.7%) of data per participant and condition were retained.

Time-frequency decomposition was accomplished with wavelet convolution using Hanning windows as tapers with a fixed time window length of 500 ms. Frequencies of interests were chosen between 1 and 30 Hz with bins of 1 Hz. Time points of interest were defined from 1000 ms pre-stimulus through stimulus onset in time bins of 100 ms. Based on Salari and Rose [18], statistical analyses concentrated on all electrodes that were positioned in frontal, central, or temporal locations (FP1, FP2, F3, F4, C3, C4, F7, F8, T7, T8, Fz, Cz, FC1, FC2, FC5, FC6, F1, F2, C1, C2, AF3, AF4, FC3, FC4, F5, F6, C5, C6, AF7, AF8, FT7, FT8, TP7, TP8, FPz, AFz, FCz). Further, in accordance with our hypotheses, power was averaged in the theta (3–7 Hz) and low beta (13–17 Hz) frequency bands.

### Statistical analyses

#### Behavioral analysis

In a first step, memory performance was investigated separately for the two sensory modalities. A stimulus was considered to be correctly recognized if participants responded that an item was certainly old or that an item was presumably old. Similarly, new items were classified as correctly identified if participants responded that an item was certainly new or that an item was presumably new. For correctly identified old items, source memory performance was computed by calculating the probability that participants were able to recall the correct picture that had accompanied the retrieved word during the encoding phase. Memory performances in the different modalities were compared by applying Student's t-test to the behavioral data where appropriate.

Reaction times for animate vs. inanimate judgments in the encoding phase were compared between subsequently remembered and forgotten stimuli in each modality and for correct and incorrect source memory responses. Differences between reaction times were investigated by applying Student's t-test where appropriate.

#### EEG analysis

To compare theta and low beta power between trials associated with remembered and forgotten words, the number of trials were balanced between the two conditions by randomly selecting trials from the condition with more trials until the trial number was equal to the condition with less trials. To test for statistical differences between the conditions, non-parametric permutation tests with a cluster-based correction for multiple comparisons were employed [37]. This approach is especially useful for EEG analyses since it does not rely on assumptions about an underlying sampling distribution but creates its own sampling distribution and was used to correct for multiple comparison in all relevant dimensions (electrode, time bin and frequency). In a first step, the membership of data in one condition (remembered or forgotten) was randomly shuffled, and t-tests were performed on each data point. This step was repeated 1000 times so that a distribution of t-values was created for each data point. Afterwards, the actual empirical t-value at each data point was compared to this random distribution of t-values. Data points that were significantly different from this random distribution (α = 0.05) and were adjacent in the time, space, or frequency dimension were summarized as a cluster and a cluster-based test statistic, i.e. the sum of t-values within each cluster, was extracted. This step was again repeated with the data being randomly permuted between conditions 1000 times, and a distribution of the cluster-based test statistic was extracted. The empirical cluster-based test statistic was finally compared to this distribution of random test statistics, with the p-value of each cluster indicating the percentage of random permutations that resulted in a greater test-statistic than the empirical one. As we hypothesized based on prior findings [18] that we would find pre-stimulus SMEs in the beta and theta frequency range with greater power before the onset of later remembered than later forgotten items, one-sided t-tests were applied to these two frequency bands.

## Results

### Behavioral results

#### Encoding task performance

Correct classification into animate/inanimate categories occurred in 90.4% (SEM ± 1%) of all cases indicating a high compliance in all participants during the encoding task.

#### Memory performance

Performance regarding both recognition and source memory were compared between written and spoken words. Written words were correctly remembered with an average probability of 67.3% (SEM ± 3.1%) and a false positive rate of 11.1% (SEM ± 1.5%). This resulted in an average sensitivity measure d' for visually presented words of 1.7 (SEM ± 0.1). Spoken words were correctly remembered with an average probability of 67.9% (SEM ± 3.2%) and a false positive rate of 11.5% (SEM ± 2.2%), resulting in a d' of 1.7 (SEM ± 0.1). Comparing memory performance between both presentation modalities did not reveal a significant difference in the probability of correct retrieval, *t*(18) = 0.31, *p* = .76, *CI* = (-0.04, 0.05). In fact, memory performance in both modalities was found to be significantly correlated across subjects (*r* = .8, *p* = .000043).

Sources of written words were correctly recalled with an average probability of 18.3% (SEM ± 3.1%) and a false alarm rate of 31.7% (SEM ± 2.3%). Similarly, sources of spoken words were recalled with an average probability of 17.4% (SEM ± 2.1%) and a false alarm rate of 31.7% (SEM ± 2.1%). Notably, the design of the recognition task with four different options for pictures plus the option to say that the source has not been remembered leads to a different chance level for each subject, depending on how often they choose the question mark. Chance level would mean that whenever a picture was chosen, each of the pictures would have equal probability to be chosen. That means if subjects performed at chance level, false alarm rates would be three times as high as recognition rates. Therefore, these results indicate that subjects performed above chance in the present source recognition task. A comparison between source memory performance for the two stimulus modalities did not reveal a significant difference, *t*(18) = -0.45, *p* = .66, *CI* = (-0.05, 0.03). Again, source memory performances for both modalities were significantly correlated across subjects, *r* = .81, *p* = .000031. Moreover, recognition memory rate and source memory rate were significantly correlated across subjects for spoken (*r* = .49, *p* = .03) and for written words (*r* = .64, *p* = .004).

#### Reaction times

Reaction times at encoding for subsequently recognized written words were slower compared to subsequently forgotten words (retrieved written words: M = 1.02 seconds, SEM ± 0.03 seconds, forgotten words: M = 0.95 seconds, SEM ± 0.03 seconds). The same was true for spoken words (retrieved: M = 1.3 seconds, SEM ± 0.03 seconds, forgotten: M = 1.26 seconds, SEM ± 0.03 seconds). A repeated-measures ANOVA revealed a significant effect for stimulus modality [*F*(1, 18) = 202.2, *p* = .000001] and memory performance [*F*(1, 18) = 53.46, *p* = .000001]. The interaction between both was not significant [*F*(1, 18) = 1.15, *p* = .297]. However, reaction times for recognition memory were significantly correlated between the modalities for successful retrieval (*r* = .83, *p* = .000012) as well as for failed retrieval (*r* = .79, *p* = .000066).

Reaction times at encoding for successful source memory associated with written words were slightly slower (M = 1.03 seconds, SEM ± 0.03 seconds) compared to failed source memory (M = 1.02 seconds, SEM ± 0.03 seconds). The converse pattern was found for spoken words (correct source memory: M = 1.29 seconds, SEM ± 0.03 seconds, incorrect source memory: M = 1.31 seconds, SEM ± 0.03 seconds). A repeated-measures ANOVA revealed a significant effect for stimulus modality [*F*(1, 18) = 203.82, *p* = .000001] but neither the memory performance [*F*(1, 18) = 0.06, *p* = .809] nor the interaction between both reached significance [*F*(1, 18) = 0.83, *p* = .376]. There was a significant correlation between the reaction times for correct source retrieval in both presentation modalities (*r* = .55, *p* = .016) as well as between reaction times of failed source retrieval (*r* = .78, *p* = .000078).

### Oscillatory power

#### Recognition memory

One participant was excluded from the analysis of recognition memory because there were less than 10 trials in two conditions. Thus, the number of participants for the following analyses was reduced to 18.

**Fig 3:**
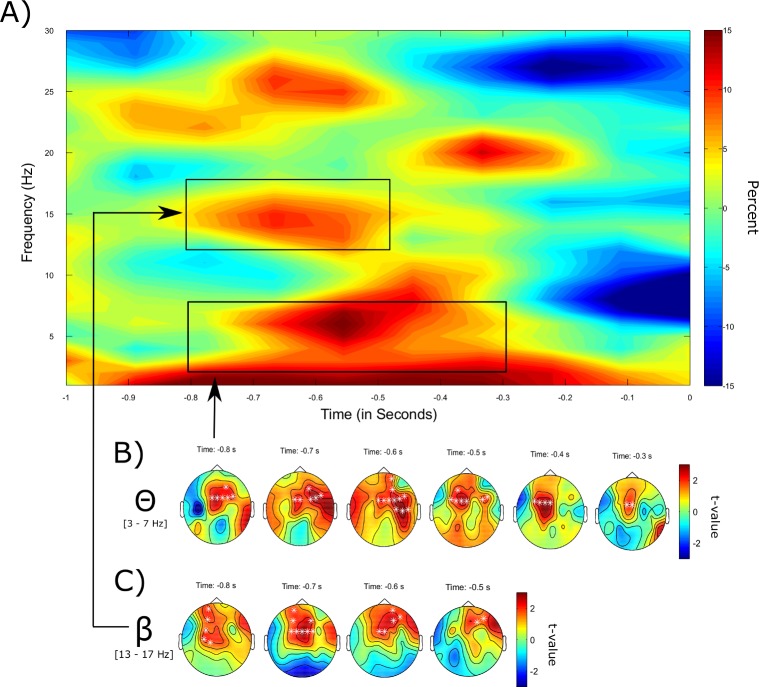
Pre-stimulus SMEs for the item memory for visually presented words. (A) Time-frequency illustration of the pre-stimulus SME for written words. Plotted is the percentage change between successful and failed retrieval averaged across all frontal, temporal, and central electrodes. (B) Depicted is the topographical distribution of the significant clusters in the theta frequency band (3–7 Hz) over time with significant data points being marked by a white * (*p* = .031, one-sided). (C) Topographical distribution of the significant clusters in the beta frequency band (13–17 Hz) over time (*p* = .01, one-sided).

First, pre-stimulus activity for visual stimuli was analyzed. For written words, the cluster-based non-parametric permutation test revealed increased power in the theta frequency range (3–7 Hz) in the pre-stimulus interval (800 to 300 ms prior to stimulus onset) associated with later remembered compared to forgotten words (*p* = .031 one-sided, corrected; see Fig 3B). The difference was found to be largest in frontal-midline areas. Additionally, in line with the hypotheses, a significant difference in the low beta frequency range (13–17 Hz) between remembered and forgotten words in the time range from -800 to -500 ms relative to stimulus onset was revealed (*p* = .01 one-sided, corrected; see Fig 3C).

The distribution of power differences between later remembered and forgotten stimuli for written words across the time and frequency domain can be seen in Fig 3A, showing that indeed the strongest effect arise in the a priori hypothesized frequency bands. Note that this figure was created for illustrative reasons only and that it was not the basis for the statistical analyses, which were applied on predefined frequency bands.

**Fig 4:**
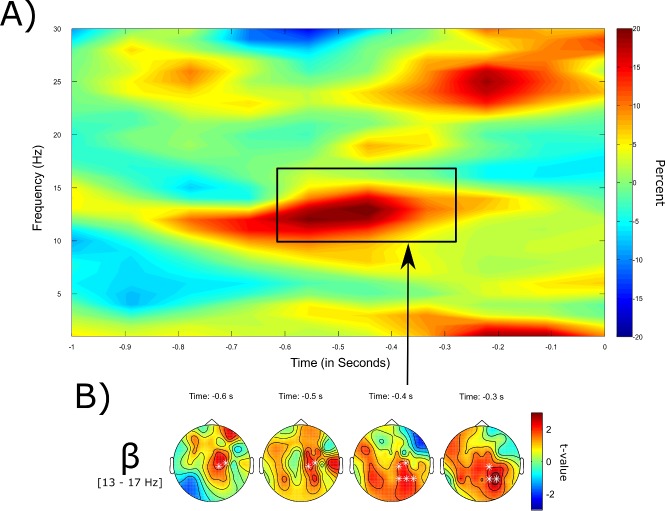
Pre-stimulus SME for aurally presented words. (A) Time-frequency illustration of the pre-stimulus SME for spoken words. Plotted is the percentage change between successful and failed retrieval averaged across all frontal, temporal, and central electrodes. (B) Depicted is the topographical distribution of the significant clusters in the low beta frequency band over time with significant data points being marked by a white * (*p* = .041, one-sided).

Next, neural pre-stimulus activity for auditory stimuli was analyzed. Similar to the results for written words, a significant subsequent memory effect in the low beta band between 600 to 300 ms before stimulus onset was revealed for the spoken words (*p* = .041, one-sided, corrected; see Fig 4B). Subsequent remembering was associated with significantly increased power in central-parietal electrodes. In contrast, no significant difference in theta power was found for this modality. The distribution of power differences between later remembered and forgotten stimuli for spoken words across the time and frequency domain can be seen in Fig 4A. Again, the figure was created after the frequency band specific analyses and did not serve as the basis for the analyses.

An investigation of the interaction between the two factors SME and modality did reveal a significant difference in theta frequencies in recognition memory 800 to 400 ms prior to stimulus onset, with a greater SME for visual than auditory stimuli (*p* = .032, two-sided, corrected). No difference in beta power was detected.

In contrast, no difference in theta power was found for the interaction analysis. The distribution of power differences between later remembered and forgotten stimuli for spoken words across the time and frequency domain can be seen in Fig 4A. Again, the figure was created after the frequency band specific analyses and did not serve as the basis for the analyses.

**Fig 5:**
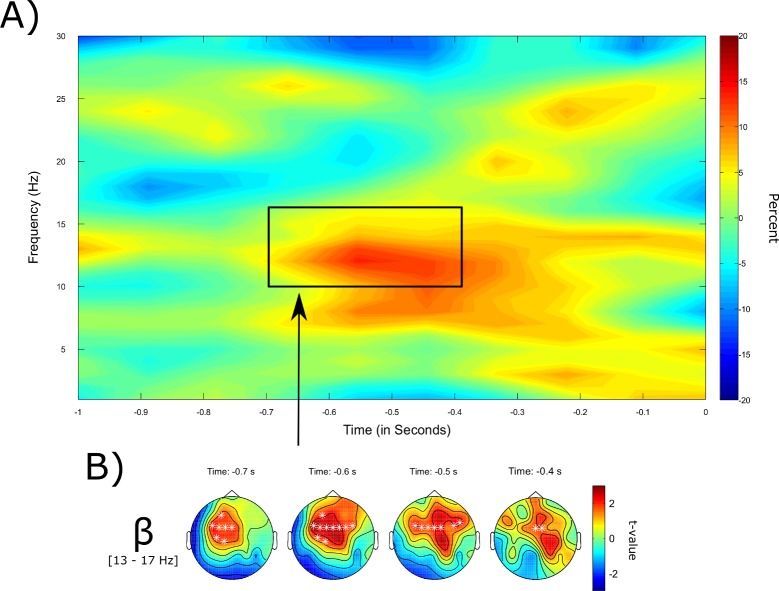
Pre-stimulus SME for words pooled across both modalities. (A) Time-frequency illustration of the pre-stimulus SME pooled across modalities. Plotted is the percentage change between successful and failed retrieval averaged across all frontal, temporal, and central electrodes. (B) Depicted is the topographical distribution of the significant clusters in the low beta frequency band over time with significant data points being marked by a white * (*p* = .041, one-sided).

Finally, pre-stimulus activity was compared across modalities between remembered and forgotten stimuli. The analysis revealed a significant difference in beta frequencies 700 to 400 ms before stimulus onset (*p* = .041, one-sided, corrected; see Fig 5B). The distribution of power differences is depicted in Fig 5A. The figure did not serve as the basis for the analyses. No such effect was revealed for theta frequencies.

#### Source memory

For analyses regarding source memory performance, seven participants were excluded from the subject pool as there were fewer than 10 trials in at least one of the conditions, reducing the number of participants to 12.

**Fig 6:**
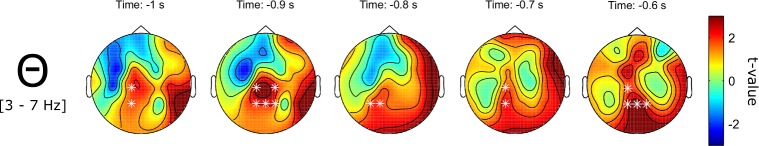
Pre-stimulus theta band (3–7 Hz) SME for context pictures associated with written words. Depicted are t-values with corrected significant data points being marked by a white * (*p* = .038, one-sided).

First, source memory for visual stimuli was analyzed. The cluster-based non-parametric permutation test revealed a significant difference between trials in which the source of a remembered written word was retrieved and trials in which the word not the source was successfully retrieved. In line with the hypotheses, source retrieval was associated with increased central-parietal theta power (3–7 Hz) before stimulus onset (-1000 to -600 ms relative to stimulus onset, *p* = .038, one-sided, corrected, see Fig 6). In contrast, no difference in beta or any other frequency band was revealed for the comparison of successful and failed source memory associated with written words.

**Fig 7:**
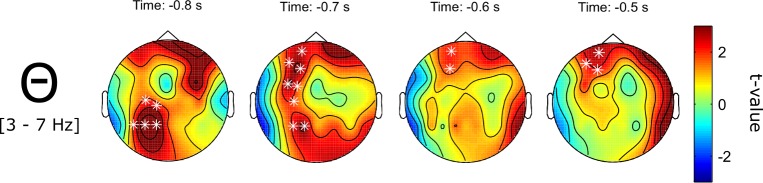
Pre-stimulus theta band (3–7 Hz) SME for context pictures associated with spoken words. Depicted are t-values with corrected significant data points being marked by a white * (*p* = .001, one-sided).

Next, source memory for auditory stimuli was analyzed. Similar to the results for written words and as expected, trials in which the source of a spoken word was successfully retrieved were characterized by an increase in theta power (3–7 Hz) in the pre-stimulus interval (800 to 500 ms before stimulus onset, *p* = .001, one-sided, corrected). The topography of the effect shows a drift over time from rather central-parietal electrodes (comparable to the topography of the source SME for written words) to frontal electrodes (see Fig 7).

Again, no effect of beta power was revealed for correct source memory in this sensory modality. Comparing pre-stimulus activity in the visual and auditory modality did not reveal any significant difference in either theta or beta frequencies.

**Fig 8:**
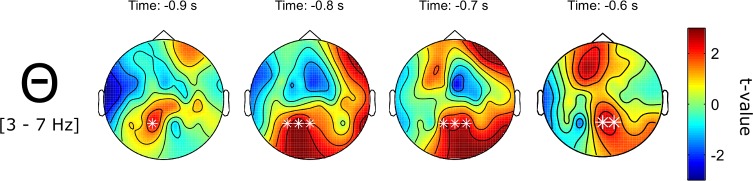
Pre-stimulus theta band (3–7 Hz) SME for context pictures associated with words pooled across both modalities. Depicted are t-values with corrected significant data points being marked by a white * (*p* = .042, one-sided).

Finally, source memory pooled across the two modalities was analyzed. As expected, a significant difference between subsequently remembered and forgotten sources in theta frequencies was found in the pre-stimulus interval between 800 to 500 ms before stimulus onset (*p* = .042, one-sided, corrected, see Fig 8). No such effect was found for beta frequencies.

## Discussion

The present study investigated the influence of stimulus modality on memory-related ongoing theta and low beta pre-stimulus oscillations in a subsequent memory paradigm using verbal material in visual and auditory sensory modalities. Further, the relationship between these oscillations and the encoding of an associated source was examined. For visually presented words only, subsequent recognition was characterized by increased pre-stimulus theta power in frontal areas, which is in line with previous findings [4, 5, 16]. Importantly, in both conditions, regardless of modality, a memory-related increase in pre-stimulus activity was revealed for activity in the low beta frequency band. The association between pre-stimulus beta power and subsequent recognition is in accordance with prior studies investigating the encoding of pictures [18, 19]. It is worth noting that both the present study and the prior study by Salari and Rose [18] investigated ongoing pre-stimulus activity in the absence of a cue announcing the upcoming stimulus. Hence, there seems to be an association between pre-stimulus low beta activity and ongoing fluctuations relevant to the ability to form new memory traces for visual stimulus material. The present study also interestingly revealed increased low beta activity to be associated with later memory for aurally presented words. In contrast, no effects in theta activity were found for this stimulus modality. Based on these results, it can be suggested that increased ongoing pre-stimulus low beta but not theta activity reflects a rather general memory-promoting state and is not restricted to processing in one single modality.

The moderating mechanism via which low beta pre-stimulus power acts on successful long-term memory encoding can so far only be inferred. One candidate mechanism is modality-independent attention. Support for this hypothesis is given by Egner and Grunzelier [38], who used lower beta frequencies as a target in a neurofeedback-training for healthy participants. By training participants to specifically increase low beta power, they were able to manipulate their attentional capacity. They concluded that enhanced beta power might increase activation in a noradrenergic vigilance and alertness network. In addition to attention, frequencies in the lower beta range (13–18 Hz) are especially involved in semantics [39], binding of stimuli in speech processing [40], and predictions of upcoming stimuli [25]. Since low beta oscillations are also responsible for maintaining the current status and signaling the appearance of new input [26, 27], it seems logical that changes in low beta power would be in some way responsible for ongoing fluctuations in the ability to encode new information into long-term memory. Low beta oscillations were also reported to play a role in inhibitory processes [22–24]. Though these studies typically associated beta oscillations with inhibition during the processing of a stimulus, inhibitory processes prior to the onset of a stimulus might prepare the memory system for the integration of new input by inhibiting competing memory traces. In accordance with this idea, Waldhauser et al. [23] found evidence for the influence of low beta oscillations on the suppression of competing memories during episodic memory retrieval. Similar, Jensen and Mazaheri [22] concluded that high alpha/low beta activity controls information processing by inhibiting task-irrelevant regions in the brain. Our results could be explained using this framework as it seems plausible that such inhibitory processes expressed by low beta oscillations are independent from the modality of the to-be-remembered stimulus. However, further research is necessary to better understand the cognitive processes underlying the memory-promoting function of ongoing beta oscillations.

It is noteworthy that even though the temporal and spectral dynamics of the pre-stimulus subsequent memory effects in the low beta band are highly similar across modalities, the topographies of the two effects indicate that there might be different underlying neural generators of the signal. Perhaps fluctuations in attentional or inhibitory processes, as potentially reflected in low beta power, occur in modality-specific brain regions. By presenting stimuli from the two modalities in a block-wise design, participants in the present study were enabled to focus their attentional capacities on the modality of interest in each block.

Information about the underlying neural structures that generate the present low beta SME would perhaps be helpful to draw further conclusions about the functionality of ongoing low beta oscillations and their role in encoding. While prior research localized the source of pre-stimulus theta SMEs in medial temporal lobe structures, such as the hippocampus [5], it would be interesting to explore whether these locations are also involved in the generation of the pre-stimulus low beta SMEs. The topographical distributions of the present results suggest the involvement of medial and frontal neural structures in the generation of memory-related low beta oscillations. To get further insight into this issue, future studies should consider applying combined EEG and fMRI measurements to track down the sources of these effects.

To investigate the influence of ongoing oscillations on qualitative aspects of encoding in the present study, correctly remembered trials were subdivided into trials with successful and unsuccessful source memory. Trials with successful source memory were characterized by increased central pre-stimulus theta activity compared to trials with unsuccessful source memory, which is in accordance with previous research investigating the highly related concepts of recollection and familiarity [31]. The present study further extends this work, as pre-stimulus theta power was also found to be increased for the successful encoding of a visually presented context picture associated with aurally presented stimuli. Consequently, it can be suggested that ongoing theta activity might reflect fluctuations in the ability to initiate deep, qualitatively demanding encoding processes, such as the binding of stimulus with context features. Crucially, this holds true even if such encoding processes depend on cross-modal integration.

The role of theta oscillations in memory formation has been discussed widely in the past decades [41–44]. Prior studies suggested that pre-stimulus theta power resembles a specific preparatory state for memory processing that is related to particular task demands like reward expectancy [16]. Therefore, elevated pre-stimulus theta power might reflect engagement in a motivational context that modulates subsequent memory retrieval. Further, our finding that pre-stimulus theta power seems to be beneficial for the encoding of the context associated with an item is highly interesting since increased theta power has previously been suggested to be involved in the binding of different kinds of information [43–45]. In a recent investigation, Hanslmayr and Staudigl [44] postulated that the role of theta oscillations during encoding might be to bind items to their contextual features and that elevated theta activity might resemble the overlap between encoding and retrieval situation. Thus, oscillations in the theta range might not be related to the encoding process itself [5, 29] but either to the linking of item-item associations [28] or to the motivational context embedded in the learning situation. Following this interpretation, it is interesting that the present study did not reveal an increase in theta power for words that were presented aurally. Whether pre-stimulus theta activity is specific to the visual modality or participants were less motivated to learn aurally presented words can only be speculated. For working memory processes, activity in the theta frequency range seems to be responsible for similar tasks, independent of sensory modality [46]. Alternatively, as proposed by Salari and Rose [18], theta activity might not have a direct functional role in recognition memory formation itself, and the lack of theta effects associated with successful auditory recognition memory might support this claim. Theta activity might indirectly be related to memory performance through a moderating mechanism that acts on more qualitative aspect of memory formation.

One limitation regarding the analysis of source memory in the current investigation is that source memory performance was found to be greater than chance performance but overall rather low. Two factors could be responsible for this result. Firstly, each source was associated with a large amount of words, which could have potentially led to interference due to source confusion. Secondly, participants were given the option not to choose one of the four scenes but also to state that they did not remember the source. Most participants preferred the option to not answer the question instead of risking a wrong answer, which is why the latter aspect might be more relevant for the current results. However, source confusion and the risk-avoiding response bias could of course be tightly related, as increased interference between source-item associations might lead to stronger insecurity and thereby promote a more conservative response behavior. Separating the confusion error and the failure to retrieve source memory is not possible from the present data. Low source memory rates entailed a reduced trial number in the correct source memory condition for the EEG analyses.

Even though the present results indicate that there is an association between beta and theta oscillations and memory formation, it cannot be concluded that these associations are actually causal. Future studies could investigate the causal role of the ongoing activity in different frequency bands in the encoding process by directly manipulating oscillatory activity through neuromodulatory approaches such as transcranial alternating current stimulation (tACS) [47], rhythmic transcranial magnetic stimulation (rTMS) [48], or neurofeedback [49] and observing associated changes in memory performance.

To conclude, the present study confirmed prior findings that ongoing fluctuations in power in the theta and low beta frequency range can be associated with the ability to encode a stimulus, with higher power in both frequency bands before stimulus onset being predictive of later memory for that stimulus. We extended the research in this field by showing that subsequent memory effects in the low beta band arise independent of stimulus modality, while theta shows specific effects for visual stimuli only. When it comes to qualitative aspects of memory formation however, increased pre-stimulus theta power was found to be associated with the ability to encode an associated context accompanying the stimulus, even in conditions when context (visual) and stimulus (visual or auditory) needed to be interlinked across different modalities. We therefore suggest that increased ongoing low beta activity reflects a memory-promoting state that is likely to be moderated by modality-independent attentional or inhibitory processes, whereas high ongoing theta power is suggested as an indicator of the enhanced binding of incoming interlinked information.
